# The Proangiogenic Capabilities of Malignant Ascites Generated by Aggressive Ovarian Tumors

**DOI:** 10.1155/2017/2592496

**Published:** 2017-09-20

**Authors:** Justyna Mikuła-Pietrasik, Paweł Uruski, Sebastian Szubert, Konstantin Maksin, Rafał Moszyński, Dariusz Szpurek, Aldona Woźniak, Stefan Sajdak, Andrzej Tykarski, Krzysztof Książek

**Affiliations:** ^1^Department of Hypertensiology, Angiology and Internal Medicine, Poznań University of Medical Sciences, Długa 1/2 Str., 61-848 Poznań, Poland; ^2^Division of Gynecological Surgery, Poznań University of Medical Sciences, Polna 33 Str., 60-535 Poznań, Poland; ^3^Department of Clinical Pathology, Poznań University of Medical Sciences, Przybyszewskiego 49 Str., 60-355 Poznań, Poland

## Abstract

Here we examined whether malignant ascites may determine ovarian tumor angiogenesis, and if so whether ascites generated by highly aggressive serous and undifferentiated cancers are more proangiogenic than those from less aggressive clear cell and endometrioid tumors. Angiogenesis was analyzed according to expression of CD31, CD34, and connexin 43. Proliferation and migration of endothelial cells were tested using fluorescence-based methods. The quantification of angiogenic agents and hypoxia-inducible factor 1*α* (HIF-1*α*) was performed using specific immunoassays. Results showed that the expression of CD31 and CD34 in serous and undifferentiated tumors was greater, whereas endothelial expression of connexin 43 was lower than in clear cell and endometrioid lesions. Serous cancers that formed in the presence of ascites displayed increased expression of connexin 43 in vascular smooth muscles as compared with tumors developed in the fluid's absence. Endothelial cells exposed to ascites from serous and undifferentiated tumors proliferated and migrated more vigorously than cells subjected to ascites from clear cell and endometrioid cancers. They also exhibited an increased level of HIF-1*α* and produced increased amounts of multiple proangiogenic agents. Our results indicate that high vascularization of aggressive ovarian tumors may be associated with profound angiogenic capabilities of ascites generated by these tumors.

## 1. Introduction

Angiogenesis, considered to be the formation of new blood vessels and/or increased permeability of existing ones, is one of the most critical steps in successful progression of primary and metastatic tumors [1]. The central element of this process is endothelial cells, which under strictly defined biochemical conditions cause a degradation of the vascular basement membrane, proliferate, and migrate into the perivascular stroma, which eventually leads to the development of tubular structures and tissue neovascularization [2].

Although the role of angiogenesis in ovarian cancer progression, including the development of intraperitoneal metastases, is indisputable [3], there are some issues that hamper gaining a full understanding of the triggers and mechanisms responsible for disease-related intensification of this process. This is the case, for example, of the role of malignant ascites and pathological fluid buildup in the abdomen which concerns a subset of patients with ovarian malignancy [4]. It is known that the formation of ascites is critically regulated by the activity of proangiogenic stimuli, particularly vascular endothelial growth factor (VEGF) [5], and that the ascites are rich in various mediators of angiogenesis, such as angiogenin, angiopoietin-2, and interleukin 8 (IL-8) [6]. On the other hand, however, the mechanisms by which the ascites modulate tumor neovascularization, including their effect on the angiogenic behavior of the vascular endothelium, are only speculative.

Taking into account the above, we designed a project based on both* in vivo* and* in vitro* analyses in which we tried to conclusively answer the three following questions: (1) Does the presence of malignant ascites increase angiogenesis within ovarian tumors? (2) Is the proangiogenic potential of ascites from highly aggressive type II ovarian tumors (serous and undifferentiated histotypes) stronger as compared with those from less aggressive type I cancers (clear cell and endometrioid histotypes)? and (3) How does the proangiogenic activity of ascites involve altered proliferation and migration of vascular endothelial cells?

## 2. Materials and Methods

### 2.1. Materials

Unless otherwise stated, all chemicals and cell culture plastics were from Sigma (St. Louis, MO, USA).

### 2.2. Patients

The study was performed on tumors excised from the peritoneal cavity from 48 women with ovarian cancer (stages IIIC and IV) who were undergoing cytoreductive surgery. The analyses included four histological subtypes of ovarian cancer, that is, clear cell (*n* = 12), endometrioid (*n* = 12), high-grade serous (*n* = 12), and undifferentiated (*n* = 12). The histopathology, grade, and stage of the tumors were assigned in accordance with the criteria of the International Federation of Gynecology and Obstetrics. The patients did not receive chemotherapy prior to surgery. The study included patients in whom tumors had developed in either the presence (*n* = 24) or absence (*n* = 24) of peritoneal ascites.

Malignant ascites were collected in sterile conditions; the fluids were centrifuged at 2500 rpm for 5 min and then cell-free supernatants were stored in aliquots at −80°C until required. Control, benign fluids were obtained from age-matched patients (*n* = 6) undergoing abdominal surgery due to the presence of noncancerous lesions, that is, cystadenoma mucinosum multiloculare. This study using tumors and ascites was approved by the bioethics committee at Poznań University of Medical Sciences (consent number 543/14, May 7 2014) and all patients gave their informed written consent.

### 2.3. Cell Cultures

EA.hy926 endothelial cells were obtained from ATCC (Rockville, MD) and cultured in DMEM medium supplemented with 10% fetal bovine serum, L-glutamine (2 mM), glucose (4500 mg/L), sodium pyruvate (110 mg/L), and antibiotics.

### 2.4. Immunohistochemistry

The tumors were fixed in 4% formalin, embedded in paraffin, and cut into 3 *μ*m sections. Deparaffinization, rehydration, and epitope retrieval were conducted using Envision Flex Target Retrieval Solution (Dako, Glostrup, Denmark). The cancerous tissue was identified using H+E staining. Tumor angiogenesis was evaluated according to immunohistochemical detection of endothelial cell markers, that is, CD31 and CD34. Briefly, the specimens were incubated with an antibody against CD31 (Leica Biosystems, Buffalo Grove, IL) and against CD34 (Santa Cruz Biotechnology, Santa Cruz, CA), both diluted 1 : 25. Mouse lung preparations were used as the positive and negative controls. The staining was performed using Autostainer Link 48 (Dako, Carpinteria, CA).

Expression of connexin 43 was performed using Connexin-43 Polyclonal Antibody (Proteintech, Manchester, UK), diluted 1 : 200. The reaction was visualized using the Novolink Polymer Detection System (Novocastra Reagents, Wetzlar, Germany).

Planimetric analysis of the brown-stained area reflecting the presence of CD31/CD34/connexin 43-positive cells was conducted using ImageJ 1.47v (Wayne Rasband, National Institute of Health, USA). Pictures of all immunoreactions were taken using an Axio Vert.A1 microscope (Carl-Zeiss, Jena, Germany).

### 2.5. Proliferation and Migration Assays

Proliferation of EA.hy926 cells exposed to 10% malignant or benign ascites (for 72 h) was examined using Cell Proliferation Kit I (PromoKine; Heidelberg, Germany). Migration of EA.hy926 towards 100% malignant ascites used as a chemoattractant (for 4 h) was quantified using ChemoTx migration chambers (Neuro Probe, Gaithersburg, MD, USA). Both assays were performed strictly according to the manufacturer's instructions.

### 2.6. Immunoassays

Concentrations of EGF, FGF2, GRO-1, HGF, IL-8, MCP-1, and VEGF were quantified in conditioned medium generated by EA.hy926 cells. Briefly, 3 × 10^5^ of cells were seeded into 25 cm^5^ flasks, allowed to attach for 4 hours, and incubated with 10% malignant and benign ascites for 72 h. Afterwards the ascites were removed and the cells were carefully washed and incubated for 72 h to generate an autologous conditioned medium. Samples of conditioned media were centrifuged, filtered through a 0.2 *μ*m pore size filter, and stored at −80°C until required. Measurement of angiogenic agents was performed using appropriate DuoSet® Immunoassay Development kits (R&D Systems, Abingdon, UK). The concentration of transcription factor HIF-1*α* in EA.hy926 cells exposed to ascites (10%, for 72 h) was determined in cell extracts using Human/Mouse Total HIF-1*α* ELISA (R&D Systems). All assays were performed strictly according to the manufacturer's instructions. Fluorescence was recorded using a Synergy™ 2 spectrofluorometer (BioTek Instruments, Winooski, VT, USA).

### 2.7. Statistics

Statistical analysis was conducted using GraphPad Prism™ 5.00 software (GraphPad Software, San Diego, USA). The means were compared with repeated measures analysis of variance (ANOVA) with the Newman-Keuls test as post hoc. When appropriate, the Mann–Whitney test was used. Results were expressed as means ± SEM. Differences with a *P* value < 0.05 were considered to be statistically significant.

## 3. Results and Discussion

What is definitely known about the relationship between the presence of malignant ascites, angiogenesis, and the progression of ovarian tumors is that the suppression of blood vessel development, for example, by neutralization of VEGF, results in a reduction of ascite formation and tumor growth [7], and that the use of anti-VEGF strategies (most often bevacizumab, but also trebananib, aflibercept, cediranib, pazopanib, sorafenib, and sunitinib) alone or in combination with classic chemotherapy (carboplatin with paclitaxel, carboplatin with gemcitabine, pegylated liposomal doxorubicin, and topotecan) improves survival rates and the patient's life quality [8]. At the same time, direct evidence for the proangiogenic capabilities of malignant ascites contributing to improved tumor development is mainly speculative, as it is solely based on the identification of an increased level of angiogenic agents in this fluid [6] or on an assessment of ascites-related angiogenesis using very artificial models. The latter is the case, for example, for a study in which the proangiogenic capacity of malignant ascites was monitored using skin angiogenesis assay in mice [9].

**Figure 1:**
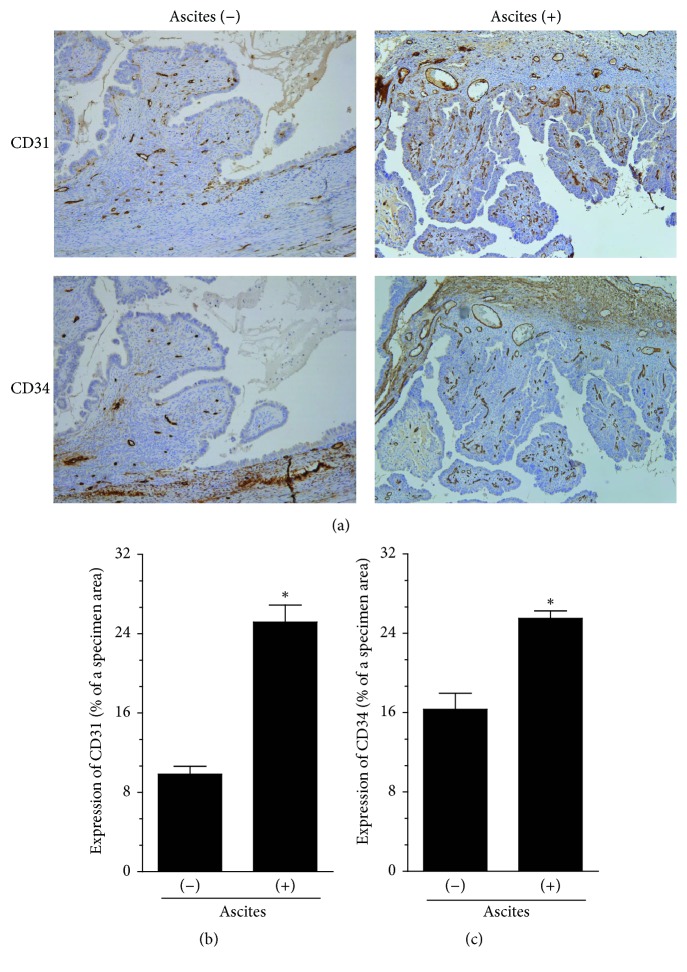
*Microscopic evaluation of angiogenesis in serous ovarian tumors that developed in either the presence or absence of malignant ascites*. The development of microvessels was analyzed according to immunohistochemical reactions against CD31 and CD34, that is, markers of endothelial cells. Panel (a) shows the representative results of CD31 and CD34 staining (the brown color indicates a positive reaction). Panels (b) and (c) show the results of the quantification of the brown-stained area reflecting the presence of CD31- (b) and CD34-positive cells (c). The results are expressed as a percentage (%), and the whole area of a specimen is considered to be 100%. ^*∗*^*P* < 0.05 versus ascites (−). The results derive from an analysis of tumors from 6 different patients per group and are expressed as mean ± SEM.

In the study presented here, we tried to address the issue of the role that malignant ascites may play in ovarian cancer neovascularization by using an optimal model, that is, samples of tumors excised during cytoreductive surgery from patients with various types of ovarian cancer and tumor histotype-specific malignant fluids. Initially, however, we used high-grade serous ovarian carcinoma, which is the most frequent type of ovarian cancer [10], as a representative model of ovarian cancer to distinguish the magnitude of angiogenesis in tumors that had developed in either the presence or absence of ascites. In experiments as presented in Figure 1 and concerning immunostaining against CD31 and CD34 antigens as markers of endothelial cells [11], we found that tumors whose formation was accompanied by ascites displayed increased density of microvessels as compared with lesions which had developed without ascitic fluid. In this regard our findings are in agreement with those of Gawrychowski et al., who worked exclusively on fluids from serous cancer and found their proangiogenic capacity [9].

**Figure 2:**
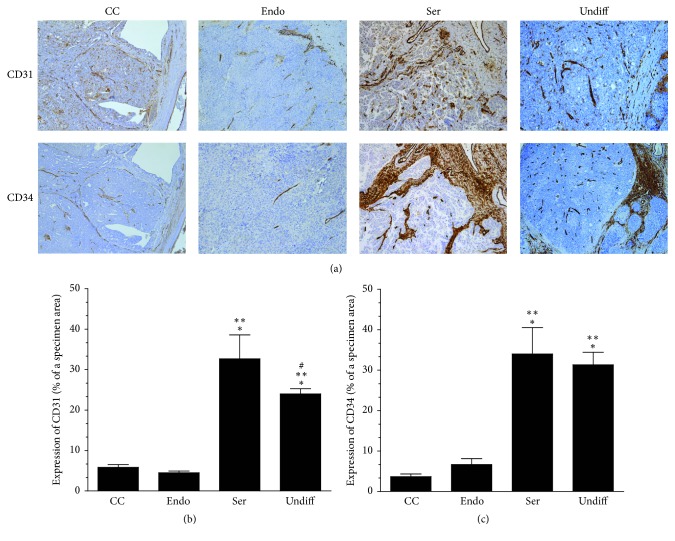
*Microscopic evaluation of angiogenesis in clear cell (CC), endometrioid (Endo), serous (Ser), and undifferentiated (Undiff) ovarian tumors that developed in the presence of malignant ascites*. Panel (a) shows the representative results of CD31 and CD34 staining (the brown color indicates a positive reaction). Panels (b) and (c) show the results of the quantification of the brown-stained area reflecting the presence of CD31- (b) and CD34-positive cells (c). The results are expressed as a percentage (%), and the whole area of a specimen is considered to be 100%. ^*∗*^*P* < 0.05 versus CC; ^*∗∗*^*P* < 0.05 versus Endo; ^#^*P* < 0.05 versus Ser. The results derive from an analysis of tumors from 6 different patients per group and are expressed as mean ± SEM.

Following this observation, we conducted analogical immunoreactions using tumors representing type I (clear cell and endometrioid) and type II (serous and undifferentiated) cancers that had developed in the presence of comparable volumes of malignant ascites and we found that tumors belonging to highly aggressive histotypes, that is, serous and undifferentiated ones [12], were characterized by significantly higher vascularization than those displaying lower aggressiveness (Figure 2).

**Figure 3:**
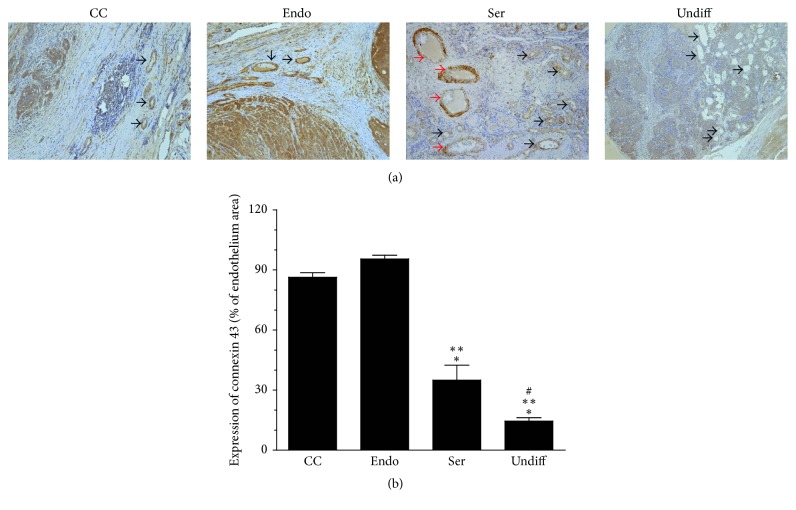
*Microscopic evaluation of the expression of connexin 43 in vascular endothelial cells within clear cell (CC), endometrioid (Endo), serous (Ser), and undifferentiated (Undiff) ovarian tumors that developed in the presence of malignant ascites*. Panel (a) shows the representative results of connexin 43 staining (the black arrows indicate a positive reaction). Note that large vessels in the serous tumors display characteristic patchy staining with areas lacking connexin 43 expression (red arrows). Panel (b) shows the results of the quantification of the brown-stained area reflecting the presence of connexin 43. The results are expressed as a percentage (%), and the whole area of an endothelium is considered to be 100%. ^*∗*^*P* < 0.05 versus CC; ^*∗∗*^*P* < 0.05 versus Endo; ^#^*P* < 0.05 versus Ser. The results derive from an analysis of tumors from 6 different patients per group and are expressed as mean ± SEM.

This effect coincided with markedly decreased expression of gap junction protein connexin 43 in the vascular endothelium within serous and undifferentiated cancers, which may suggest that the ascites also stimulated the second (besides increased motility of the endothelial cells) critical aspect of angiogenesis, that is, increased permeability of existing blood vessels [13] (Figure 3). It is worth noting that in tumors of serous origin, but not in the remaining cancer histotypes, the large blood vessels exhibited characteristic patchy expression of endothelial connexin 43, which means that despite this protein's relatively high expression they also possessed significant areas that were completely lacking this junctional protein with probably conspicuous permeability (see the red arrows in Figure 3(a)).

**Figure 4:**
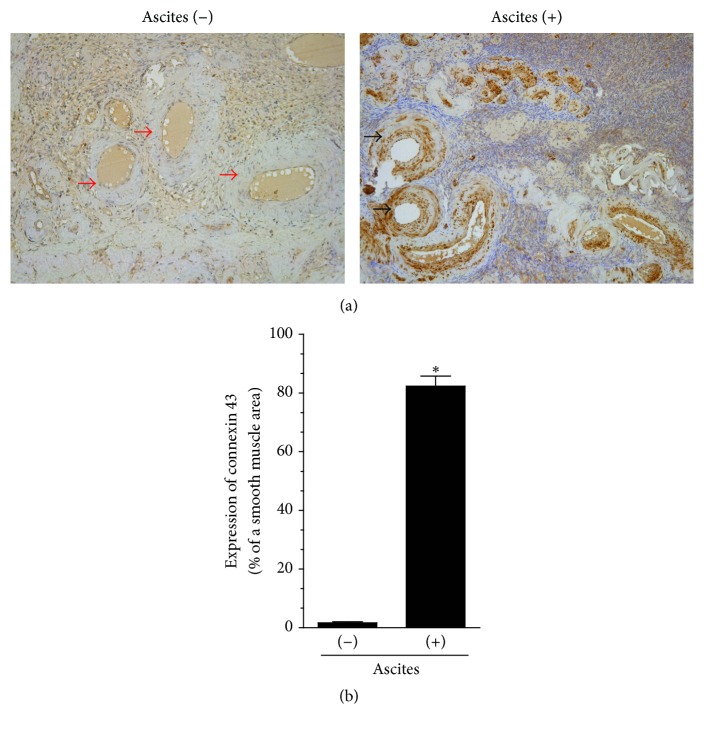
*Microscopic evaluation of the expression of connexin 43 in vascular smooth muscle cells within serous ovarian tumors that developed in either the presence or absence of malignant ascites*. Panel (a) shows the representative results of connexin 43 staining (the black arrows indicate a positive reaction; the red arrows indicate a negative reaction). Panel (b) shows the results of the quantification of the brown-stained area reflecting the presence of connexin 43. The results are expressed as a percentage (%), and the whole area of the vascular smooth muscle cells is considered to be 100%. ^*∗*^*P* < 0.05 versus ascites (−). The results derive from an analysis of tumors from 6 different patients per group and are expressed as mean ± SEM.

Unexpectedly, measurements of connexin 43 in the same cancer histotype but within vascular smooth muscle cells revealed that expression of the protein was very high in the presence of malignant ascites, whereas connexin 43 was almost undetectable in the absence of the ascites (Figure 4). This effect, which was found exclusively in the serous tumors (the remaining histotypes displayed relatively weak and comparable staining), may suggest a functional transformation of smooth muscle cells, in particular their switch from a contractile to a synthetic phenotype [14]. This may be, in turn, more evidence for the proangiogenic properties of malignant ascites, as vascular smooth muscle cells with synthetic characteristics are known to display increased ability to proliferate and migrate [15].

**Figure 5:**
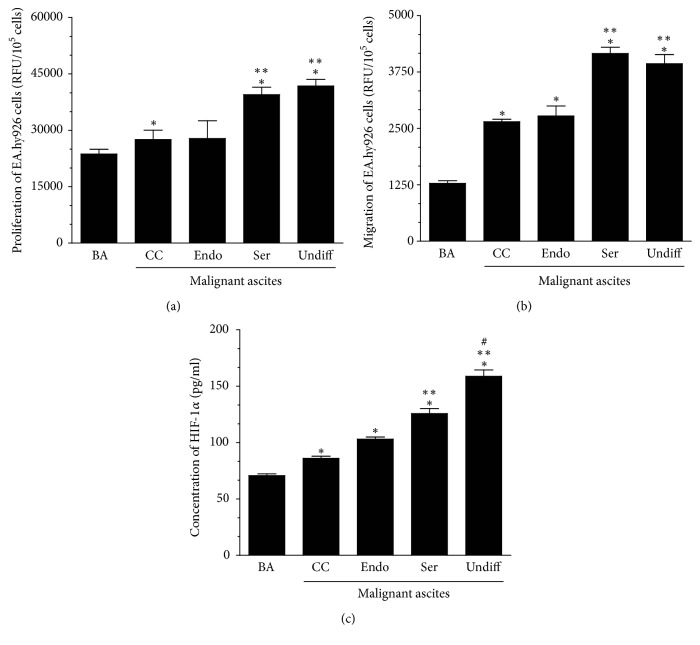
*Effect of malignant ascites from clear cell (CC), endometrioid (Endo), serous (Ser), and undifferentiated (Undiff) ovarian tumors on the proliferation (a) and migration (b) of EA.hy926 endothelial cells, and on the concentration of transcription factor HIF-1α in these cells (c)*. ^*∗*^*P* < 0.05 versus BA; ^*∗∗*^*P* < 0.05 versus CC/Endo; ^#^*P* < 0.05 versus Ser. The experiments were performed with both malignant and benign ascites obtained from 6 patients per group. The endothelial cells were used in quadruplicates. The results are expressed as mean ± SEM. BA: benign ascites; RFU: relative fluorescence units.

Having phenomenologically established that malignant ascites seem to promote angiogenesis within ovarian tumors and that ascites from serous and undifferentiated histotypes act more proangiogenically than ascites from clear cell and endometrioid tumors, we then decided to analyze the mechanistic aspects of these phenomena by using an* in vitro* model. Experiments with EA.hy926 endothelial cells, which are commonly used in studies on angiogenesis [16], showed that ascites obtained from patients with serous and undifferentiated ovarian tumors stimulated either proliferation or migration of the cells more efficiently than ascites from clear cell and endometrioid tumors (Figures 5(a) and 5(b)). All malignant ascites, in turn, fueled the motility of endothelial cells significantly stronger than benign fluids obtained from noncancerous patients. This comparison, which is often used in studies on ascitic fluids [17], may implicate that ascites formed during cancer development differ significantly from those whose accumulation accompanies nononcological diseases.

It is possible that the predominance of fluids derived from aggressive cancers is associated with their biochemical composition, in particular with an increased concentration of soluble proangiogenic stimuli, including VEGF, TGF-*β*1, HGF, SDF-1, IL-8, CXCL5, GRO-1, and MCP-1 [18]. This would be in line with reports pointing to increased progression of more aggressive ovarian cancer histotypes associated with an upregulated level of specific mediators of a given element of cancer cell progression in malignant ascites [17]. In addition, it cannot be excluded that this improved motility of endothelial cells may also be the result of some phenotypic rearrangements in the endothelial cells, in particular the functional switch towards the synthetic profile [15].

Furthermore, we found that malignant ascites from serous and undifferentiated tumors alter the secretory profile of endothelial cells; that is, they trigger the overproduction of such angiogenic agents as EGF, FGF2, GRO-1, HGF, IL-8, MCP-1, and VEGF (Table 1). This may indicate that, apart from direct stimulation of angiogenesis by proteins present in the ascites, angiogenic reactions of the vascular endothelium elicited by the ascites may also be intensified in an autocrine manner [19]. At the same time, one cannot forget that angiogenic reactions of endothelial cells usually proceed through autocrine and paracrine loops, and that the contribution of both the mechanisms depends on a specific environmental and molecular context [20, 21].

It is worth noting that the secretion of five out of seven of the tested proteins was the highest in response to ascites generated by undifferentiated tumors, which may be another factor explaining the very high aggressiveness and the worst prognosis in patients suffering from this type of disease [22]. It should also be emphasized that the multiplicity of proangiogenic agents present in ascites as well as those hypersecreted by stimulated endothelial cells provides evidence that the proangiogenic microenvironment within the peritoneal cavity, including milky spots with a high density of blood vessels [23], is created not only by resident mesothelial cells [24].

Another type of activity of the malignant ascites discovered in this study was their effect on the concentration of hypoxia-inducible factor 1*α* (HIF-1*α*). This molecule's level was the highest in endothelial cells subjected to ascites from undifferentiated tumors, whereas it was considerably lower in cells exposed to ascites from clear cell and endometrioid tumors (Figure 5(c)). Taking into account the fact that HIF-1*α* is upregulated under hypoxic conditions characterizing the tumor environment and that the production of above listed proangiogenic agents is known to depend on this factor [25], it seems reasonable to suppose that ascites-related ovarian tumor neovascularization, including improved angiogenic reactions of endothelial cells, is transcriptionally controlled by this agent. Also in this case it should be recalled that poor prognosis of several kinds of tumors has already been correlated with the high activity of HIF-1*α* [26], which is probably causatively linked with their therapeutic resistance due to the oxygen dependency of ionizing radiation-related cytotoxicity [27].

## 4. Conclusions

Taken together, our findings indicate that augmented angiogenesis may be an important determinant of the high aggression of serous and undifferentiated ovarian tumors, and that the diversified neovascularization level of both more and less aggressive cancer histotypes may be associated with the presence and activity of malignant ascites. From a clinical point of view, these findings may additionally show the necessity of therapeutic strategies aimed at targeting ascites, particularly in patients with highly aggressive peritoneal tumors.

## Figures and Tables

**Table 1:** Concentration of soluble proangiogenic agents in conditioned medium harvested from endothelial cells exposed to benign peritoneal ascites and to malignant ascites from patients with clear cell, endometrioid, serous, and undifferentiated ovarian cancer.

Soluble factor (pg/10^5^ cells)	Benign fluids	Type I cancers		Type II cancers	
		Clear cell	Endometrioid	Serous	Undifferentiated
EGF	7.1 ± 0.8	10.9 ± 0.2^*∗*^	10.6 ± 0.3^*∗*^	16.1 ± 0.4^*∗*,a,b^	21.5 ± 0.9^*∗*,a,b,c^
FGF2	21.3 ± 0.6	18.2 ± 0.4^*∗*^	18.5 ± 0.4^*∗*^	24.5 ± 0.4^*∗*,a,b^	32.8 ± 1.0^*∗*,a,b,c^
GRO-1	201 ± 3	211 ± 9	221 ± 3^*∗*^	251 ± 3^*∗*,a,b^	299 ± 5^*∗*,a,b,c^
HGF	133 ± 1	106 ± 2^*∗*^	109 ± 7^*∗*^	152 ± 2^*∗*,a,b^	131 ± 3^a,b,c^
IL-8	125 ± 3	129 ± 3^*∗*^	123 ± 3^*∗*^	127 ± 2^*∗*^	157 ± 2^*∗*,a,b,c^
MCP-1	21 ± 1	13 ± 1^*∗*^	9 ± 1^*∗*^	58 ± 6^*∗*,a,b^	66 ± 3^*∗*,a,b^
VEGF	111 ± 3	89 ± 3^*∗*^	130 ± 4^*∗*^	178 ± 2^*∗*,a,b^	256 ± 3^*∗*,a,b,c^

The results derive from an analysis of tumors from 8 different patients per group and are expressed as mean ± SEM. ^*∗*^*P* < 0.05 versus benign fluids; ^a^*P* < 0.05 versus clear cell; ^b^*P* < 0.05 versus endometrioid; ^c^*P* < 0.05 versus serous.
